# Analysis of treatment pattern of anti-dementia medications in newly diagnosed Alzheimer’s dementia using OMOP CDM

**DOI:** 10.1038/s41598-022-08595-1

**Published:** 2022-03-15

**Authors:** JungHyun Byun, Dong Yun Lee, Chang-Won Jeong, Yerim Kim, Hak Young Rhee, Ki Won Moon, Jeongwon Heo, Yoonki Hong, Woo Jin Kim, Seung-Joo Nam, Hoon Sung Choi, Ji In Park, In Kook Chun, So Hyeon Bak, Kyoungyul Lee, Gi Hwan Byeon, Kyoung Lae Kim, Jeong-Ah Kim, Young Joo Park, Jeong Hyun Kim, Eun ju Lee, Sang-Ah Lee, Sung Ok Kwon, Sang-Won Park, Payam Hosseinzadeh Kasani, Jung-Kyeom Kim, Yeshin Kim, Seongheon Kim, Jae-Won Jang

**Affiliations:** 1 Healthcare Bigdata Section, Health research office, Radiation health institution, Korea Hydro & Nuclear Power Co, Ltd, Seongnam, Korea; 2 Department of Biomedical Informatics, Ajou University School of Medicine, Suwon, Korea; 3 Medical Convergence Research Center, Wonkwang University, Iksan, Korea; 4 Smart Business Team in Information Management Office, Wonkwang University Hospital, Iksan, Korea; 5 Department of Neurology, Kangdong Sacred Heart Hospital, Hallym University College of Medicine, Seoul, Korea; 6 Department of Neurology, Kyung Hee University Hospital at Gangdong, College of Medicine Kyung Hee University, Seoul, Korea; 7 Kangwon National University School of Medicine, Chuncheon, Korea; 8 Department of Internal Medicine, Kangwon National University Hospital, Chuncheon, Korea; 9 Department of Nuclear Medicine, Kangwon National University Hospital, Chuncheon, Korea; 10 Department of Radiology, Asan Medical Center, University of Ulsan College of Medicine, Seoul, Korea; 11 Department of Pathology, Kangwon National University Hospital, Chuncheon, Korea; 12 Department of Psychiatry, Kangwon National University Hospital, Chuncheon, Korea; 13 Department of Opthalmology, Kangwon National University Hospital, Chuncheon, Korea; 14 Department of Urology, Kangwon National University Hospital, Chuncheon, Korea; 15 Department of Environmental Health Center, Kangwon National University Hospital, Chuncheon, Korea; 16 Department of Preventive Medicine, Kangwon National University School of Medicine, Chuncheon, Korea; 17 Department of Medical Bigdata Convergence, Kangwon National University, Chuncheon, Korea; 18 Department of Neurology, Kangwon National University Hospital, Chuncheon, Korea

**Keywords:** Medical research, Epidemiology, Dementia, Alzheimer's disease

## Abstract

Anti-dementia medications are widely prescribed to patients with Alzheimer’s dementia (AD) in South Korea. This study investigated the pattern of medical management in newly diagnosed patients with AD using a standardized data format—the Observational Medical Outcome Partnership Common Data Model from five hospitals. We examined the anti-dementia treatment patterns from datasets that comprise > 5 million patients during 2009–2019. The medication utility information was analyzed with respect to treatment trends and persistence across 11 years. Among the 8653 patients with newly diagnosed AD, donepezil was the most commonly prescribed anti-dementia medication (4218; 48.75%), followed by memantine (1565; 18.09%), rivastigmine (1777; 8.98%), and galantamine (494; 5.71%). The rising prescription trend during observation period was found only with donepezil. The treatment pathways for the three cholinesterase inhibitors combined with *N*-methyl-d-aspartate receptor antagonist were different according to the drugs (19.6%; donepezil; 28.1%; rivastigmine, and 17.2%; galantamine). A 12-month persistence analysis showed values of approximately 50% for donepezil and memantine and approximately 40% for rivastigmine and galantamine. There were differences in the prescribing pattern and persistence among anti-dementia medications from database using the Observational Medical Outcome Partnership Common Data Model on the Federated E-health Big Data for Evidence Renovation Network platform in Korea.

## Introduction

Alzheimer’s dementia (AD) is characterized by a cognitive decline resulting in the loss of independence and has a significant amount of impact on the patients, caregivers, communities, and national health-care systems^1–3^. The number of patients with dementia is expected to double every 20 years and has reached approximately 40 million worldwide in 2020 due to increasing global aging population^4^. Therefore, studies concerning the treatment and interventions for dementia are increasingly important.

The Observational Medical Outcomes Partnership (OMOP) Common Data Model (CDM) is a semantic and logical data model that standardizes heterogeneous data sources into a common data format. Diverse data sources can be standardized and integrated by the CDM-based vocabulary, which allows researchers to analyze large-scale data for various clinical research. The Observational Health Data Sciences and Informatics (OHDSI) organization provides open-source solutions to use large-scale observational health data for various clinical research^5^. The OHDSI’s OMOP CDM has been adopted in pharmacoepidemiologic and pharmacovigilance research^6,7^.

The aim of this study was to assess the treatment pattern of anti-dementia medication in AD using CDM at multiple institutions. The treatment pathways as the ordered sequence of medications were analyzed using OHDSI’s large, diverse population to provide insight into clinical practice to identify the variations and patterns in AD treatment among multiple centers.

## Methods

### Data sources

This study included approximately 5 million patient-based retrospective cohort data spanning 11 years across five hospitals (Kangwon National University Hospital, Ajou University Hospital, Wonkwang University Hospital, Kangdong Sacred Heart Hospital, and Kangdong Kyung Hee University Hospital), which was then converted to OMOP CDM. This included standardized data with the same structure to obtain network-wide results through distributed research networks using the same analysis program among collaborating organizations^8,9^.

The OHDSI is an international collaborative consortium aimed at facilitating the generation of high-quality evidence by generating and applying open-source data analysis solutions to a large network of health databases worldwide, while supporting and updating the OMOP CDM database^5,10^. Most Korean hospitals use an electronic health record (EHR) system; however, numerous Korean codes for diagnosis, drugs, and procedures are not compatible with those of the international coding systems. Since 2016, data from Korean Ajou University and the Korean nationwide cohort database have been successfully transformed into the OMOP CDM model and validated^8,11^. Recently, the EHR data from 37 hospitals with 50 million patients were converted to CDM, which is easily accessible by the Federated E-health Big Data for Evidence Renovation Network in Korea (FEEDER-NET), a bio-health big data platform supported by the Korean National Project (https://feedernet.com), for collaborating OHDSI networks. Among the 37 hospitals, 14 hospitals formulated a contract as a research border-free zone, corresponding to a research free zone for multi-institution distribution of big data research. Institutions affiliated with the research border-free zone provide the same level of CDM research rights that is permitted to in-hospital researchers by those at other institutions, and the Institutional Review Board (IRB) approval of the principal investigator’s institution will be applied to other institutions. The visualized characteristics of the five databases with OMOP CDM version 5.3 are summarized in Table 1. This study was performed in accordance with the relevant guidelines and regulations of the IRB of the Kangwon National University Hospital (KNUH) and was approved by the KNUH IRB (approval No. KNUH-B-2020-12-004). As this was an observational study with de-identified data source, the requirement for informed consent was waived by the KNUH IRB.

**Table 1:** Data source descriptions.

Abbreviation	Hospital	Description	Patients, millions
KWMC	Kangwon University Medcal Center	Electronic health record from a Korean general hospital with 608 patients beds with monthly updated common data model database	0.5
AJOUMC	Ajou University Medical Center	Electronic health record from a Korean tertiary hospital with 1,096 patients beds daily updated common data model database	2.7
WKUH	Wonkwang University Hospital	Electronic health record from a Korean tertiary hospital with 798 patients beds with weekly updated common data model database	0.8
KDH	Kangdong Sacred Heart Hospital	Electronic health record from a Korean general hospital with 618 patients beds	1.1
KHNMC	Kyung Hee University Hospital At Gangdong	Electronic health record from a Korean general hospital with 654 patients beds monthly updated common data model database	0.7

### Study design and cohort definition

**Figure 1:**
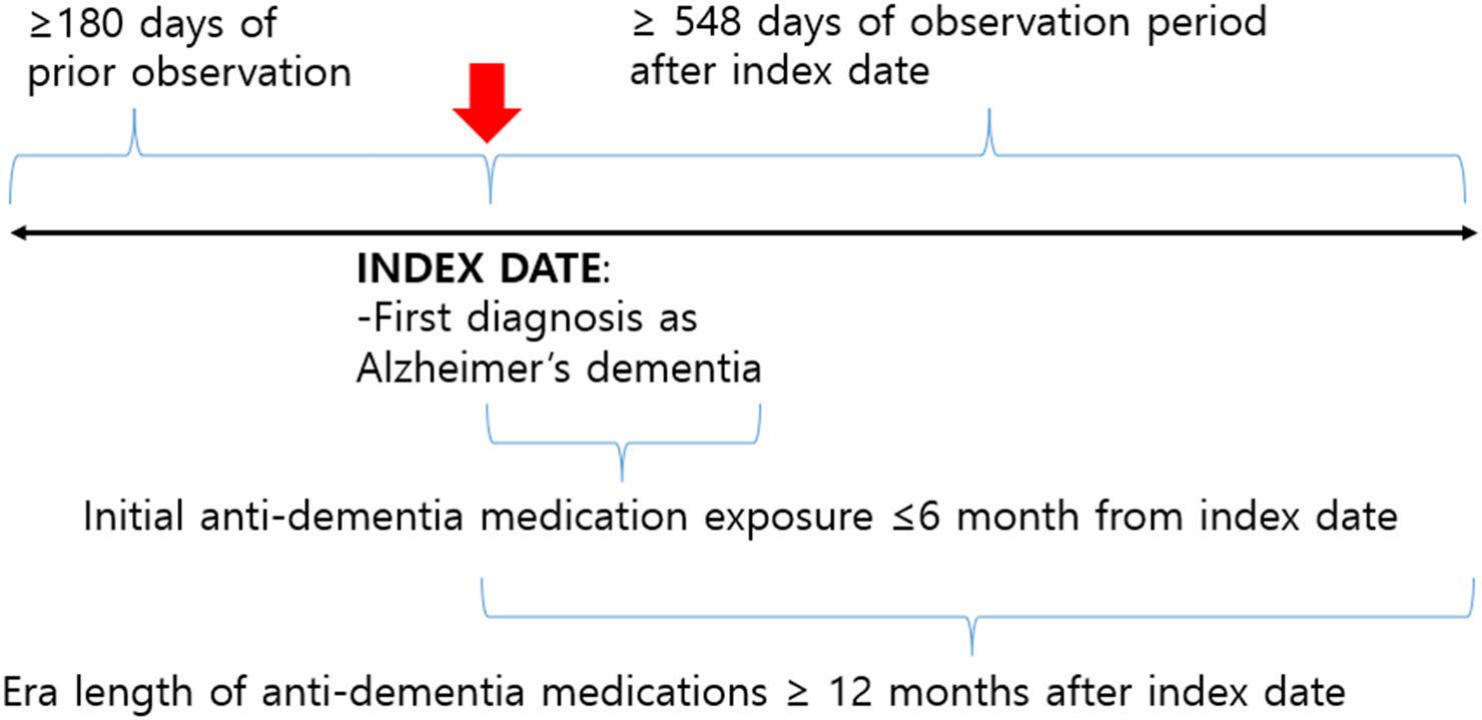
Event flow of treatment pathway. The index date for each case was the time of the first diagnosis as Alzheimer’s dementia. The patient had to have been observed for at least 180 days before and 548 days after index date. The patient had to have at least one day exposure to one of the anti-dementia medications within 6 month from index date.

We conducted a retrospective, observational, cohort study of all outpatients with AD aged over 60 years between January 1, 2009, and December 31, 2019. AD cohorts were restricted to those who were newly diagnosed and prescribed anti-dementia medications for at least 1 day within 6 months from the initial AD diagnosis. The medications included donepezil, galantamine, rivastigmine, and memantine. The diagnosis code was defined according to the Systematized Nomenclature of Medicine^12^ and was mapped to other terminologies including the International Classification of Diseases, Ninth Revision, Clinical Modification^13^. The medications were defined according to their ingredients using the RxNorm terminology^14^ and were grouped according to the classification hierarchies such as the Anatomical Therapeutic Chemical classification^15^ and First Data Bank’s terminology^16^. The index date was defined as the day of AD diagnosis in each cohort. We included patients within an observational period of over 2 years, comprising 6 months before and 1.5 years after the index date in our database (Fig. 1).

### Statistical analysis and treatment pathway

**Figure 2:**
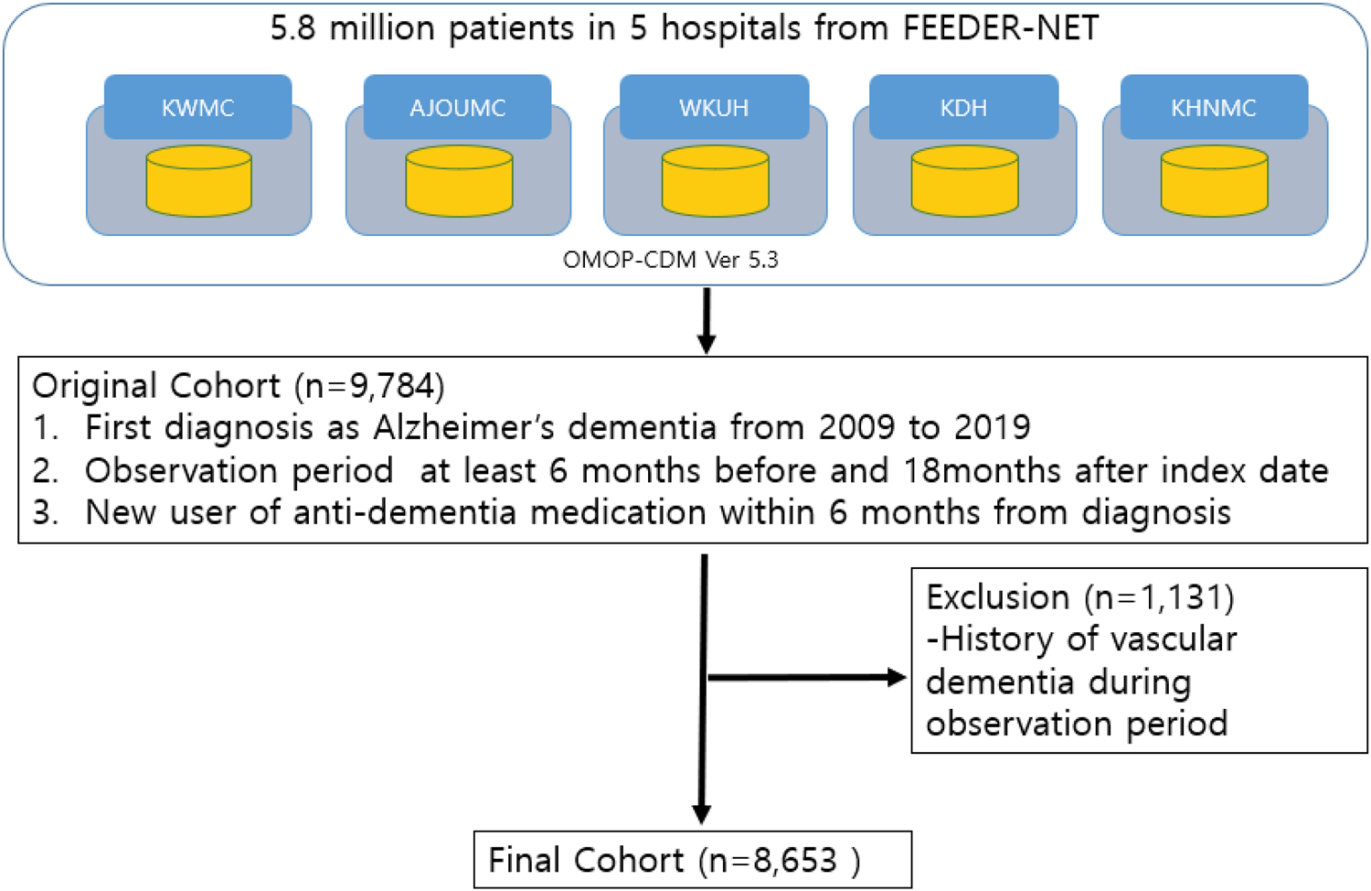
Study population. The data was analyzed as Observational Medical Outcomes Partnership Common Data Model (OMOP-CDM) with KWMC; Kangwon University Medcal Center, AJOUMC; Ajou University Medical Center, WKUH; Wonkwang University Hospital, KDH; Kangdong Sacred Heart Hospital, KHNMC; Kyung Hee University Hospital at Gangdong, OMOP-CDM; Observational Medical Outcomes Partnership Common Data Model.

OMOP CDM version 5.3 was used in this study, which included the OMOP analysis tools on the ATLAS interactive analysis platform. ATLAS version 2.7.6 and FEEDER-NET were used. Data are presented as mean ± standard deviation for normally distributed continuous variables and as numbers (percentages) for categorical variables. The sequence of medications taken by each patient was extracted from the databases, ordering them by first exposure to the medication. Note that only the first exposure was recorded in patients who switched from one medication and then returned to it. The sequence does not distinguish between switching and adding medications. Sequences were limited to two medications that are acetylcholinesterase inhibitors (AChEIs)-donepezil, galantamine, and a rivastigmine and *N*-methyl-d-aspartate receptor (NMDAR) antagonist, memantine. We then counted the number of individuals within the database who each had an observed sequence. We created tabular and graphical summaries of the sequence results, stratifying by database. We performed the analyses on five databases (Fig. 2). Among the 9784 patients with AD in the original cohort, 1131 patients with mixed AD or vascular dementia were excluded and the final cohort included 8653 patients with AD. Sunburst plots were generated from medication sequences (using software written in Hypertext Markup Language 5 and JavaScript using Data-Driven Documents, available at OHDSI.org). Persistence was defined by a continuous sequence of refills of the same anti-dementia medication^17^.

## Results

From the databases of five hospitals, 8653 patients with AD were included in this study. Detailed characteristics of each cohort population are shown in Table 2. Of the 8653 patients, 4218 received donepezil, 777 rivastigmine, 494 galantamine, and 1565 memantine within 6 months from the first AD diagnosis. Females constituted 64.4% of the participants, and the mean age of the participants was 76.4 years. The average observation time per person was 4.6 years.

**Table 2:** Demographic of cohort population.

Anti-dementia drugs	Cohort population			
	n (%)*	Female, n (%)	Mean age at drug exposure, year (SD)	Observation time, year (SD)
Donepezil	4218 (48.75)	2769 (65.6)	77.0 (8.0)	4.4 (2.3)
Rivastigmine	777 (8.98)	499 (64.2)	66.0 (7.5)	4.5 (2.4)
Galantamine	494 (5.71)	278 (56.3)	73.6 (9.0)	6.3 (3.0)
Memantine	1565 (18.09)	1042 (66.6)	76.3 (9.0)	4.4 (2.2)
Total AD	8653^†^ (100)	5569 (64.4)	76.4 (8.2)	4.6 (2.4)

n indicates the number of subjects. *Percent values indicate the number of anti-dementia medications over total number of firstly diagnosed as Alzheimer’s dementia (AD). ^†^Total number of AD subjects are not equal to the sum of each drug because there are AD patients without any medications such as *N*-methyl-d-aspartate (NMDA) receptor antagonist (e.g. Memantine) and cholinesterase inhibitors (AChEIs) (e.g. Donepezil, Rivastigmine and Galantamine). The anti-dementia drugs were prescribed within 6 months after diagnosis of AD and initial dual therapy between AChEIs and memantine was possible.

**Figure 3:**
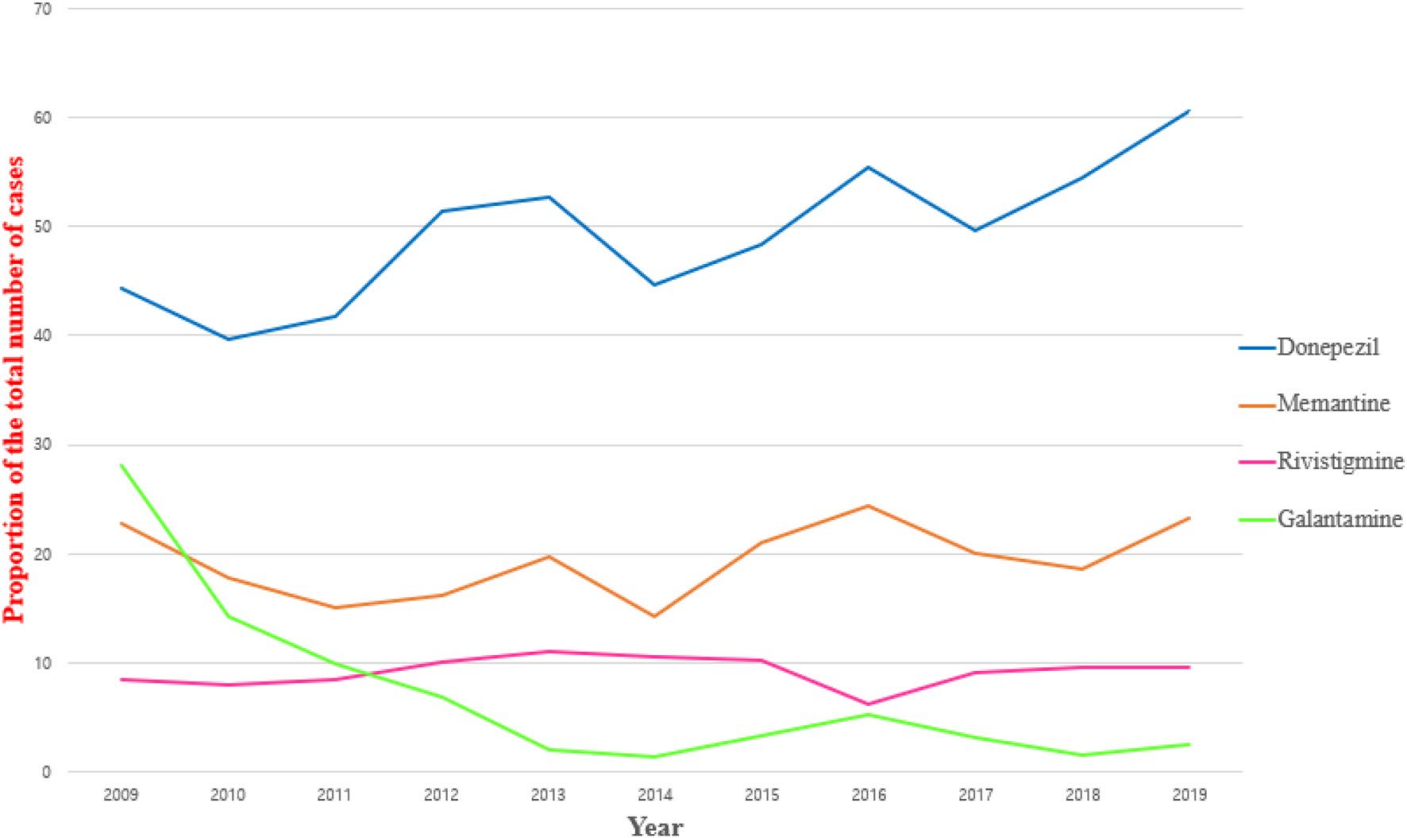
Trends for anti-dementia drug treatment of Alzheimer’s disease as broken line graphs. The horizontal axis represents the year and the vertical axis represents the proportion of the total number of cases.

Some trends related to anti-dementia medications are presented as lines in Fig. 3. Among the three AChEIs, only donepezil showed an increasing trend from 44.3% in 2009 to 60.5% in 2019. The percentage of patients on rivastigmine was approximately 10% from 2009 to 2019. The falling trend of galantamine was observed from 28.2% in 2009 to 2.5% in 2019. Furthermore, the downward trend was more obvious after 2010. The percentage of patients on the NMDA antagonist memantine was 22.8% in 2009 and 23.3% in 2019.

**Figure 4:**
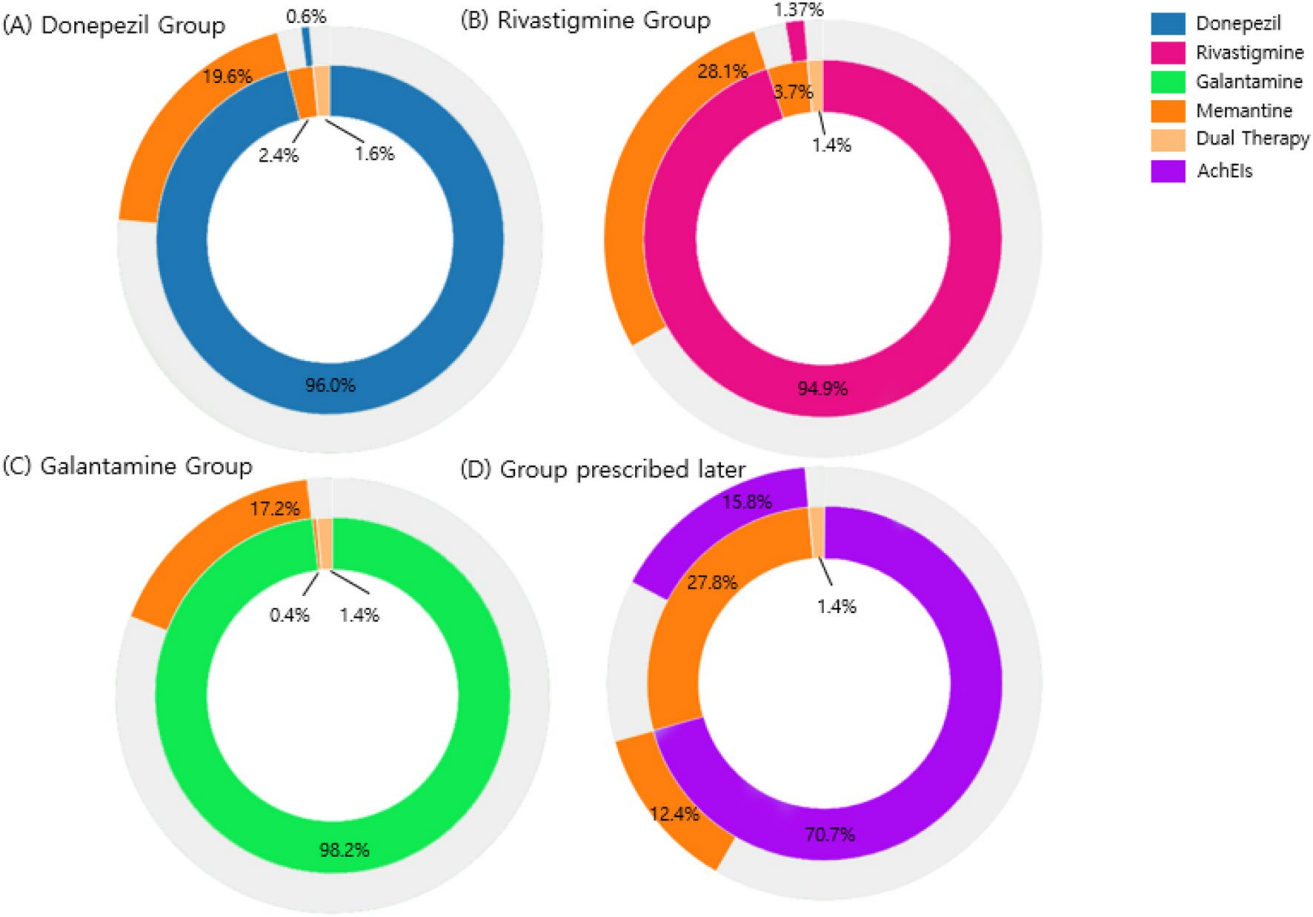
Treatment pathway of each choline esterase inhibitor (AChEI) group combined with NMDA receptor antagonist from all hospitals. For each choline esterase inhibitor group, Donepezil (**A**), rivastigmine (**B**), galantamine (**C**) and Group prescribed drugs at least 6 months later from initial diagnosis (**D**), the inner circle shows the first medication that the patient took, the second circle revealed the second medication.

The treatment pathways for the three AChEIs in combination with an NMDA antagonist are illustrated in Fig. 4. In the donepezil group, 96.0% and 19.6% of patients were prescribed donepezil and memantine as the first and second medication, respectively, whereas in the rivastigmine group, 94.9% and 28.1% of patients were prescribed rivastigmine and memantine as the first and second medication, respectively. In the galantamine group, 98.2% and 17.2% were prescribed galantamine and memantine as the first and second medication, respectively. Of the patients who did not use any drug initially but were prescribed medication/s at least 6 months after the initial diagnosis, 70.7% and 12.4% of them were prescribed AChEIs and memantine as the first and second medication, respectively. The administration rate of dual therapy with AChEIs in combination with memantine was as low as 1.4–1.6% for each of the previous groups.

**Figure 5:**
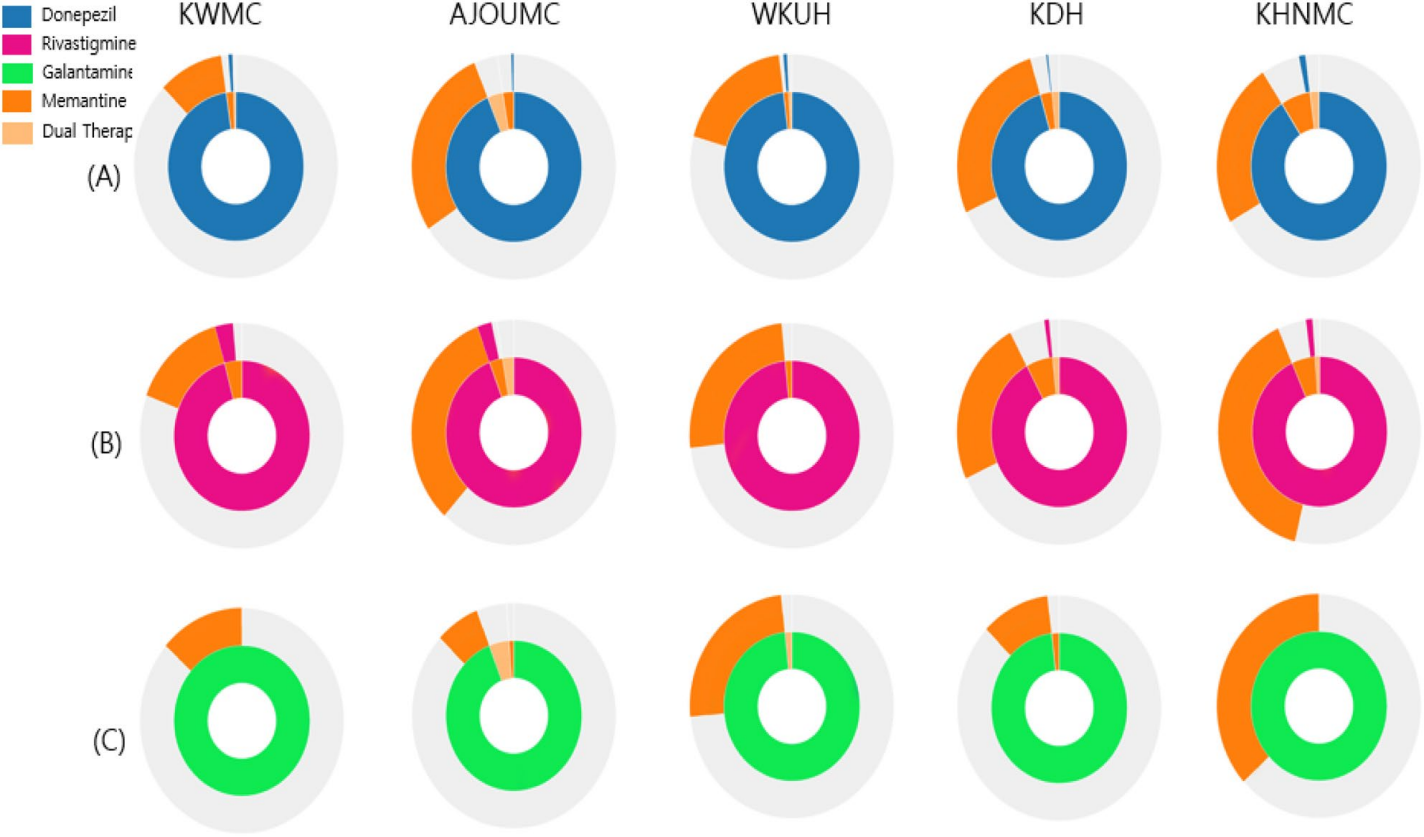
Treatment pathway for each choline esterase inhibitor group combined with NMDA receptor antagonist from database of each hospital. Donepezil (**A**), rivastigmine (**B**) and galantamine (**C**), the inner circle shows the first medication that the patient took, the second circle revealed the second medication.

Figure 5 revealed treatment pathways for all hospitals to illustrate heterogeneity. The use of memantine as combination therapy on top of AChEIs was quite rare across all hospitals.

**Figure 6:**
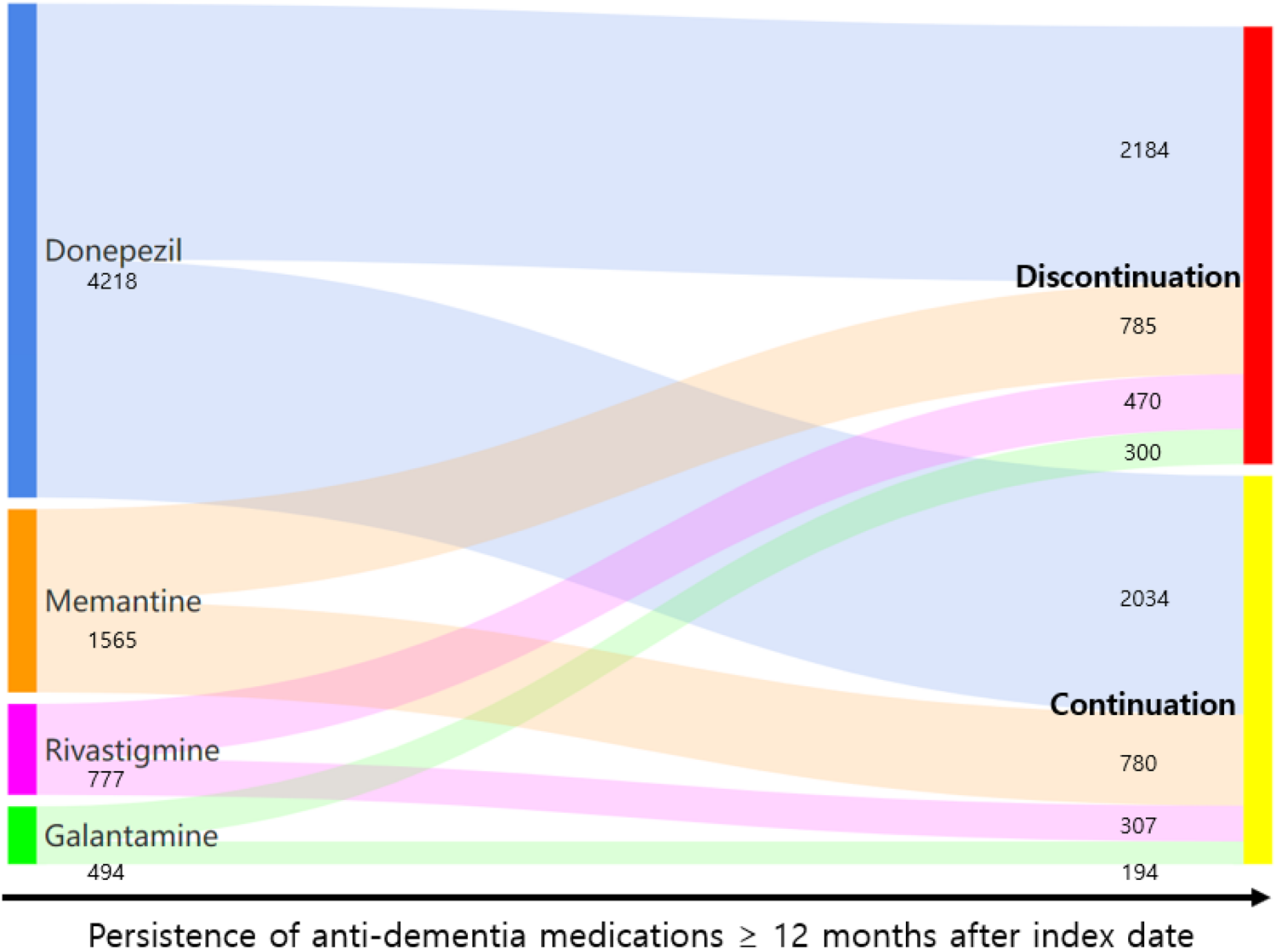
The drug continuation of each anti-dementia medication over 12 months.

Figure 6 shows the proportion of each anti-dementia medication used over 3, 6, and 12 months. Donepezil and memantine showed a similar trend, with persistence of approximately 60% over 6 months and 50% over 12 months. Similarly, rivastigmine and galantamine showed a similar trend with persistence of approximately 50% over 6 months and 40% over 12 months.

## Discussion

In this study, we demonstrated trends of anti-dementia medications using databases converted to OMOP CDM from five hospitals for 11 years. The total number of patients included those originating from two tertiary hospitals and three general hospitals and denotes the actual number, rather than a statistical estimate of a limited sample. The most important finding of this study is that the persistence rate of anti-dementia medications are similar to those previously reported^18,19^. To the best of our knowledge, this is the first study that investigated the treatment trends for AD using OMOP CDM, a standard format for medical and health data.

The increasing trend in prescribing anti-dementia medications was found only with donepezil (Fig. 3) which was also confirmed by a recent study that used the National Health Insurance Service (NHIS) claims data from 2008 to 2019^20^. This increasing trend in the usage of donepezil might partly be due to the increasing prevalence and incidence of dementia, which is in line with the recent analysis using the NHIS Senior Cohort data^21^. Another reason could be due to the Korean National Dementia Plan, a government initiated bundle of programs^22,23^ initiated in 2012 that includes the Voucher Program for Dementia Treatment to help patients with AD. Despite the benefits of this program, there are some concerns regarding the increasing false-positive diagnosis of AD^22^. The difference in the reimbursement criteria could be another explanation for the difference in trend for each drug. Among the AChEIs, donepezil has the widest range of the indications for its use and the reimbursement criteria cover its use for mild-to-severe AD, whereas galantamine has indications to be used in only mild-to-moderate AD^24^. Although the indications for rivastigmine use is also restricted to mild-to-moderate AD, the rivastigmine patch is allowed to be used for mild-to-severe AD. Memantine, an NMDAR antagonist, is indicated for moderate-to-severe AD and shows a similar graph pattern with donepezil, although the percentage of patients was stably preserved across the observation period.

The treatment pathway between the AChEIs and NMDAR antagonist was also analyzed and demonstrated a high rate for the AChEIs as the first medication and a low rate for the NMDAR antagonist either as a monotherapy or dual therapy, although the rate as the second medication was relatively high (Fig. 4). This trend remained similar across each hospital with some differences in the detailed rate of NMDAR antagonist use as the second medication (Fig. 5). This trend might be related to the treatment guidelines for AD^25,26^ as well as the reimbursement criteria of the Korean National Health Insurance^24^. Previous meta-analyses have revealed that AChEIs delay cognitive dysfunction and decline in performance of daily activities for 6–12 months on average and slow the decline in global clinical dementia rating^27,28^; therefore, they are recommended for use from the initial stages of AD^29,30^. Meanwhile, memantine is indicated by the Food and Drug Administration for moderate-to-severe AD^31^ as it has shown to improve scores of cognition, global function, activities of daily living, and neuropsychiatric symptoms^32,33^. According to these evidence-based recommendations, AChEIs should be prescribed at the time of diagnosis and NMDAR antagonist can be considered for patients with moderate-to-severe AD. Additionally, this treatment pathway might also be influenced by the 2-year observation period that was at least 180 and 548 days before and after the index date in our study, respectively. The pre-index observation period of 6 months was set to confirm patients with newly diagnosed AD. Some of the previous studies for the treatment pathway using the OMOP CDM database adopted the 1-year pre-index to a 3-year post index period^34^ to ensure sufficient time in identifying the pathway, whereas another study adopted a 6-month pre-index to a 1-year post index period to increase the total number of patients^35^. Despite the diversity of medical data sources from five different hospitals, a similar prescription pattern for anti-dementia medication was easily identified using the OMOP CDM database.

In our study, the rate of persistence was similar for donepezil and memantine, whereas it was higher for rivastigmine and galantamine than that in previous studies^36–38^ (Fig. 6). Discontinuation of drugs might be due to the side effects such as nausea, vomiting, anorexia, diarrhea, bradycardia, dizziness, myalgia, and insomnia caused by AChEIs^39^ as well as agitation, somnolence, headache, dizziness, confusion, hypertension, and imbalance caused by the NMDAR antagonist^32^. A recent prospective study identified a notably low discontinuation rate of 20.9% for donepezil^40^, and this difference might be derived from the study design: prospective or retrospective. Although a prospective study has some strength related to the quality of data by controlling covariates, a retrospective study, on the other hand, could save time and cost to collect data. Notably, the value of real-world data based on large number of patients is increasing as they include a wide and unrestricted population with few exclusions that can produce more generalizable data collection^41^. Contrary to our study, some studies using administrative claims data have demonstrated a longer persistence rate for galantamine than donepezil^42,43^ or similar persistence^19,44^, whereas rivastigmine showed a shorter persistence rate than donepezil in most studies^42,45,46^. This difference might also be due to the differences in the reimbursement criteria or treatment guidelines as described above.

There are several limitations in this study. First, obtaining detailed clinical information by medical record review was not possible due to the de-identified databases of CDM to secure privacy of patients. Therefore, additional information on whether the cause of discontinuation of the anti-dementia medications was due to death, follow-up failure, switching of drugs, or side effect-mediated stopping is not available in our study. Second, the OMOP CDM database does not include important information such as cognitive status represented by the index score, educational level, and genetic status, which are important conventional factors. Lastly, the conversion of electronic medical records to CDM database is prone to have innate data quality issues. Therefore, erroneous results can be derived from inadequately mapped code in diagnosis, drug exposure, and outcomes. Despite these limitations, our study demonstrated CDM-based long-term trends in anti-dementia treatment and this finding can be easily extrapolated to the OHDSI network hospitals through the FEEDER-NET platform in Korea.

While the drug utilization pattern can also be analyzed by claims data such as the NHIS^20^, there are several notable differences. First, the time and resources required for the investigation of drug utilization patterns are far lesser for the OMOP CDM database than for the NHIS. Ethics approval from the researchers’ IRB requires the submission of a study proposal, which should be reviewed by the NHIS review committee before providing data^47,48^. This review process usually takes over 3–4 months after the initial application for the NHIS data due to the increasing number of requests from investigators. Moreover, the fee for data usage is also considered necessary^48^. However, it is faster and easier for access to the database of multi-institutional OMOP CDM by the FEEDER-NET platform as it does not need a long-term review process or fee for data usage. Additionally, the OMOP CDM database has advantages in terms of the intuitive analysis tool such as ATLAS, whereas the NHIS data is analyzed by coding-based software such as R or SAS.

In conclusion, comparing the trends in treatment of anti-dementia medications, only donepezil showed an increasing trend, whereas galantamine showed a decreasing trend. Additionally, donepezil and memantine were associated with a longer persistence rate than rivastigmine and galantamine. This suggests that donepezil is better tolerated, while differences in reimbursement criteria should be considered.

This study provided largely consistent results, but some heterogeneity was observed using the OMOP CDM database on the FEEDER-NET platform from five hospitals. With more institutions on the research platform, the scope of the study and understanding in treatment variation will increase covering a broader population.

## Data Availability

CDM data are designed to support a distributed research network. Thus, access to the data is restricted on internal private networks. Therefore, data are not publicly available.
